# Blackbird: structural variant detection using synthetic and low-coverage long-reads

**DOI:** 10.1093/bioadv/vbaf151

**Published:** 2025-07-04

**Authors:** Dmitry Meleshko, Rui Yang, Salil Maharjan, David C Danko, Anton Korobeynikov, Iman Hajirasouliha

**Affiliations:** Tri-Institutional PhD Program in Computational Biology and Medicine, Weill Cornell Medical College, New York, NY 10021, United States; Institute for Computational Biomedicine, Department of Physiology and Biophysics, Weill Cornell Medicine of Cornell University, New York, NY 10021, United States; Tri-Institutional PhD Program in Computational Biology and Medicine, Weill Cornell Medical College, New York, NY 10021, United States; Institute for Computational Biomedicine, Department of Physiology and Biophysics, Weill Cornell Medicine of Cornell University, New York, NY 10021, United States; Tri-Institutional PhD Program in Computational Biology and Medicine, Weill Cornell Medical College, New York, NY 10021, United States; Institute for Computational Biomedicine, Department of Physiology and Biophysics, Weill Cornell Medicine of Cornell University, New York, NY 10021, United States; Independent Researcher, St. Petersburg, Russia; Institute for Computational Biomedicine, Department of Physiology and Biophysics, Weill Cornell Medicine of Cornell University, New York, NY 10021, United States; Englander Institute for Precision Medicine, Weill Cornell Medicine of Cornell University, New York, NY 10021, United States

## Abstract

**Motivation:**

Recent benchmarks show that most structural variations, especially within 50–10,000 bp range cannot be resolved with short-read sequencing, but long-read structural variant callers perform better on the same datasets. However, high-coverage long-read sequencing is costly and requires substantial input DNA. Reducing coverage lowers cost but significantly impacts the performance of existing structural variation (SV) callers. Synthetic long-read technologies offer long-range information at lower cost, but leveraging them for SVs under 50 kbp remains challenging.

**Results:**

We propose a novel hybrid alignment- and local-assembly-based algorithm, Blackbird, that uses synthetic long reads and low-coverage long reads to improve structural variant detection. Instead of relying on whole-genome assembly, Blackbird uses a sliding window approach and synthetic long-read barcode information to assemble local segments, integrating long reads to improve structural variant detection accuracy. We evaluated Blackbird on real human genome datasets. On the HG002 Genome in a Bottle (GIAB) benchmark, Blackbird in hybrid mode demonstrated results comparable to state-of-the-art long-read tools, while using less long-read coverage. Blackbird requires only 5× coverage to achieve F1-scores (0.835 and 0.808 for deletions and insertions) similar to PBSV and Sniffles2 using 10× PacBio Hi-Fi long-read coverage.

**Availability and implementation:**

Blackbird is available at https://github.com/1dayac/Blackbird.

## 1 Introduction

Structural variations (SVs) greatly contribute to human genome diversity (Feuk *et al.* 2006). Characterizing the full spectrum of SVs in human genomes makes it possible to understand the distribution of SVs in different populations (Collins *et al.* 2020). Additionally, SVs have been found to play a critical role in the development of certain diseases, such as cancer (Li *et al.* 2020) and Alzheimer’s (Wang *et al.* 2022). SVs are typically referred to any genome sequence altering event longer than 50 bp (Abecasis *et al.* 2010). SVs can be classified into two categories: (i) large-scale events affecting tens of thousands of bases in the genome, such as translocations, large inversions, and segmental duplications, and (ii) smaller events, less than 10 kbp in size, which can be classified as insertions, deletions, or other types. Notable examples of the latter class include mobile element insertions and deletions. Both classes of SVs have been previously studied using various sequencing technologies and algorithms. Currently, the vast majority of whole-genome sequencing is performed using standard short-read technologies. While SVs are biologically and clinically important, their identification remains challenging. Short-read methods have low sensitivity and can introduce false-positive calls due to their relatively short fragment lengths and lack of long-range information. (Treangen and Salzberg 2012, Chaisson *et al.* 2019). In particular, prediction of SV events inside or around repetitive regions using short reads is very challenging. All available SV calling tools are based on the assumption that read mapping to the reference genome is correct, however, in a typical sequencing experiment, less than 80% of the reads are mapped unambiguously to the reference genome. For ambiguous reads originating from repeat sequences, often only one random or best hit is considered, which makes accurate SV prediction extremely challenging (Treangen and Salzberg 2012). Due to the low-cost of short-read sequencing, however, multiple tools such as SVaBa (Wala *et al.* 2018), DELLY (Rausch *et al.* 2012), LUMPY (Layer *et al.* 2014), Manta (Chen *et al.* 2016), GRIDSS (Cameron *et al.* 2017), Tardis (Hormozdiari *et al.* 2011), Cue (Popic *et al.* 2023) and others are developed and being used for genome-wide SV calling. These methods use different signatures of short-read alignments to the reference genome in order to characterize SV events. For example, DELLY uses read-pair and split-read signatures to identify SV breakpoints. LUMPY utilizes split-read, read-pair, and prior knowledge about SVs (e.g. a database of common SVs) and combines these signals into a probabilistic framework to calculate the probability of novel breakpoint for a given position. Manta combines uses and split-read evidence during SV discovery, but does not require split-reads or successful breakpoint assemblies to report a variant in cases of strong evidence. SVaBa uses a local assembly approach, that allows calling insertions with higher sensitivity. Cue uses a deep learning-based approach to call and genotype SVs (Popic *et al.* 2023). Cue encodes various SV signals from read alignments into 2D images and uses a neural network for SV detection.

Recent technology advancements, specially increases in accuracy and decreases in cost of long-read platforms (PacBio, Oxford Nanopore) promise to overcome some of the limitations of short reads. Long-read methods such as PBSV (Pacific Biosciences 2018), SMRT-SV (Chaisson *et al.* 2015, Huddleston and Eichler 2016), NextSV (Fang *et al.* 2018), Sniffles2 (Smolka *et al.* 2024) or SVIM (Heller and Vingron 2019) can effectively identify most SV types, including long insertions, by aligning long reads to the reference genome and subsequently sub-assembling regions with breakpoint signatures. However, high-coverage long-read sequencing may still be more expensive compared to short-read methods. Additionally, long reads require high-quality and high-input DNA. One way to offset the cost of SV calling using long-read sequencing is to lower the sequence coverage. However, existing tools typically experience a rapid decline in recall rates when coverage falls below a certain threshold (Jiang *et al.* 2021).

Recently introduced low-cost and low-input synthetic long-read (SLR) sequencing technologies—such as single tube long fragment read (stLFR) (Wang *et al.* 2019) and UST’s Transposase Enzyme-Linked Long-read Sequencing (TELL-Seq) (Zheng *et al.* 2016) provide long-range information useful for characterizing genomes and metagenomes. In these new technologies, input DNA is first sheared into long fragments of 5–100 kbp. After shearing, a barcode is ligated to short reads from these fragments, ensuring that short reads originating from the same fragment share the same barcode. Finally, the short reads are sequenced using standard sequencing technologies (e.g., Illumina HiSeq).

Despite their potential, SLR methods have not gained widespread popularity. This is partly due to a lack of sensitive, robust, and scalable methods that leverage SLR technology. To date, only a few studies have attempted to compare SLR with conventional short-read or long-read methods for SV detection (Meleshko *et al.* 2022, Popic *et al.* 2023).

Existing SLR-specific SV detection methods such as NAIBR (Elyanow *et al.* 2018), Linked-SV (Fang *et al.* 2018), or GROC-SVs (Spies *et al.* 2017) utilize barcode similarity between distant parts of the target genome, therefore, they are able to call only large events. Given that SLR platforms provide only shallow coverage of long fragments per barcode, it is challenging to detect smaller events using barcode-specific information, since it is hard to distinguish between intra-molecule gap between consecutive short reads and real SV event. An alternative approach for genome-wide SV detection is whole-genome *de novo* assembly, although it is less sensitive and computationally very expensive (Zhang *et al.* 2020).

We recently developed Novel-X (Meleshko *et al.* 2022), a tool for calling novel sequence insertions from SLR sequencing data that shows large improvement over short-read methods. Novel sequences are DNA sequences present in a sequenced sample but missing in the reference genome. Novel-X utilizes barcode information through an assembly of barcode lists originating from the same region. Contrary to the existing short-read methods (Hajirasouliha *et al.* 2010, Kehr *et al.* 2017, Kavak *et al.* 2017), results were highly concordant with SMRT-SV insertion calls from PacBio long-read data. Novel-X was designed only for novel insertion calling and is not able to call deletions and a wide range of non-novel insertions (e.g. repeat elements).

The development of new algorithms that integrate different types of sequencing data is still in its early stages. In particular, only a few algorithms effectively combine SLR and long-read sequencing data (Bayega *et al.* 2020, Adam *et al.* 2022). Here we introduce Blackbird, a novel algorithm for general SV calling that uses a combination of SLR and low-coverage long-read sequencing data (hybrid mode). Blackbird provides an SLR-only mode as well. Blackbird is based on the principle of dividing the target genome into small segments, assembling each segment separately using barcode information encoded in the aligned SLRs, and identifying SVs within each segment.

Our novel algorithmic contribution in this paper is two-fold. First, we use interconnecting barcode assembly in SLR data to solve a challenging problem of insertions and deletions calling in the 50–10 000 bp range. In addition, we implement an assembly graph-based framework to integrate low-coverage long-reads with SLR data to further improve SV detection.

## 2 Methods

**Figure 1.**
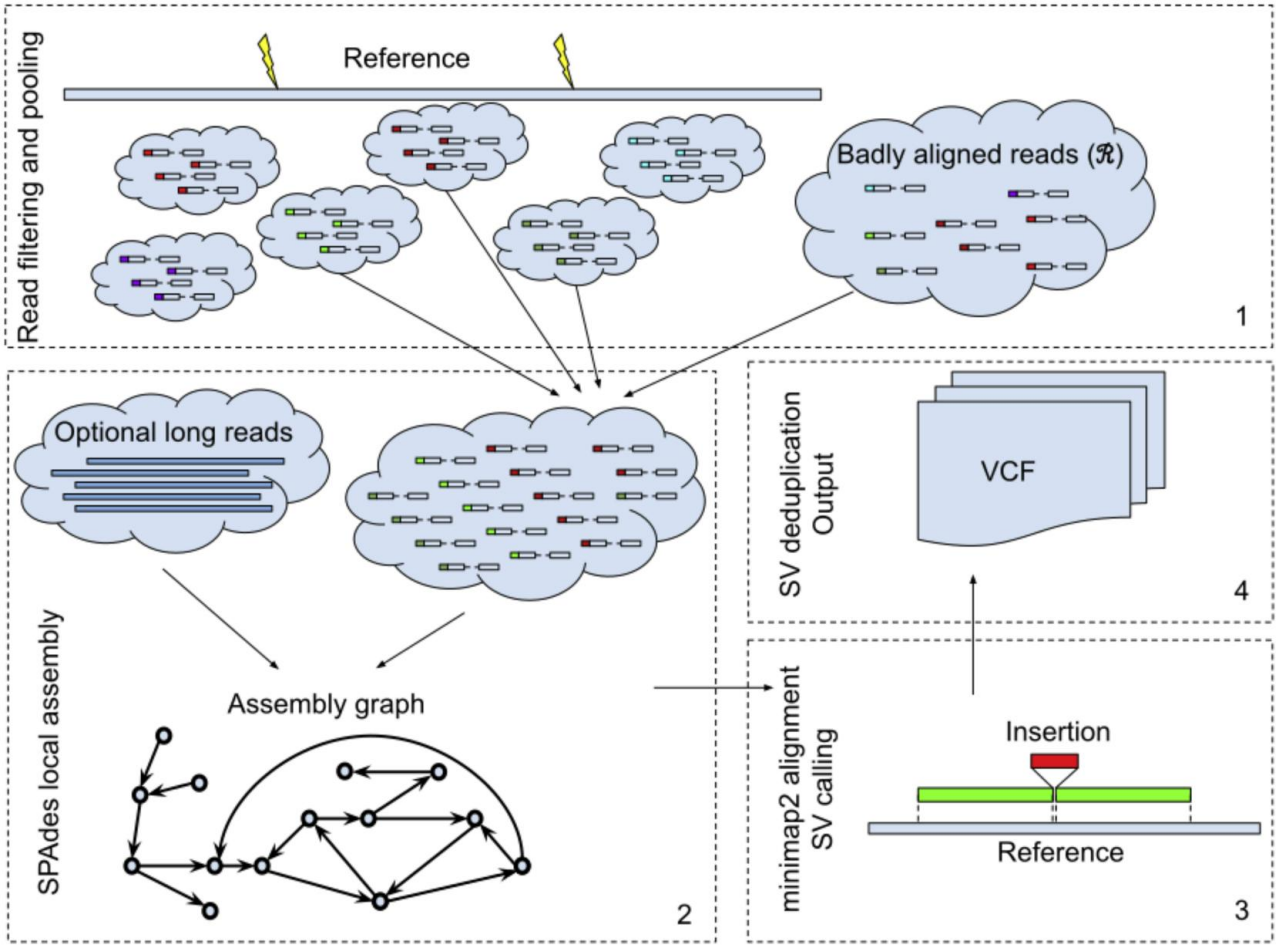
Blackbird outline. 1. The reference genome is split into segments. Inside each segment, we select a set of barcodes and collect reads with these barcodes, both aligned into the segment and unaligned. 2. Local assembly is performed for the segment, and add long-reads aligned to the segment are added if they are available. 3. The assembled contigs are mapped back to the reference genome segment using minimap2 (Li 2018), and variants are called from the alignments’ CIGAR strings. 4. SV calls are sorted, deduplicated, and stored in the VCF format.

The main steps implemented in Blackbird are presented in Fig. 1. Below, we describe each step of our method in more detail.

Blackbird calls a modified version of SPAdes assembler as a subroutine, leveraging its utility functions for local assembly. Its primary innovation is a novel approach to efficient processing and filtering, along with the strategic use of barcode information to effectively combine SLR data with long reads.

The input for Blackbird consists of a reference genome (e.g. GRCh38) and an indexed, position-sorted BAM file generated by an aligner that preserves barcode information (we utilize the BX tag of the SAM record for this purpose). We recommend using LongRanger (Marks *et al.* 2019) for this task. It is essential for users to employ the same reference genome for both read alignment and as input for Blackbird to ensure consistent results.

### 2.1 Filtering badly aligned reads

First, we search the BAM file for *badly aligned reads* to create a badly aligned read set R. R consists of badly aligned read pairs, that are defined as follows: (a) both reads in pair have a Phred quality higher than 20 (less than 1% error) for all bases, (b) at least one of the reads is not aligned or aligned to unplaced reference contigs or has more than 20% of soft-clipped bases, or (c) one of the reads has a zero value of AM tag meaning that this read cannot be clustered with other reads with the same barcode. Condition (a) is important because we do not want to keep reads of bad quality, which were not ideally aligned because of multiple sequencing errors. Conditions (b) and (c) usually arise when reads that were sequenced around breakpoint positions, repetitive regions, and from genome segments missing from the reference genome. We store R in a parallel flat hash map data structure that we refer to as read map M. M internal structure allows to efficiently querying of a subset of reads from R that have a barcode from a given barcode list B. We refer to a subset of such reads as M(B).

### 2.2 Local assembly

Then we define a set of segments S over the reference genome. By default, each segment is 50 kbp long with consecutive segments overlapping by 10 kbp. This overlap is essential for detecting SVs that span segment boundaries. Without overlap, such events might be missed. Conversely, events shorter than 10 kbp will be contained within a single segment and can be detected. As some events may be detected in multiple consecutive segments, we apply a deduplication procedure in later stages to remove redundant calls.

The choice of segment overlap and width parameters is crucial, as it influences the trade-off between assembly complexity and the size of deletions that Blackbird can detect. Our default parameters are designed to minimize the number of repeats in each segment. The length of segment overlap establishes an upper bound on the size of deletions that can be identified; if a deleted sequence completely encompasses the segment overlap, it will not be detected by our algorithm. However, since the majority of SVs are shorter than 10 kbp (Chaisson *et al.* 2019), including notable examples like Alu and L1 insertions and deletions, a segment overlap length of 10 kbp is a reasonable default choice. In this way, we are handling the most challenging classes of insertions and deletions. These parameters can, however, be modified by the user before running the tool if preferred.

At this step, our goal is to assemble one or more contigs that represent the segment, incorporating potential SVs for each segment. During the assembly process, we aim to utilize reads sequenced specifically from this segment while minimizing the inclusion of reads from other segments that may have mapped to it spuriously. For each segment *s* we define a barcode list B(s). We want to add barcode *b* to B(s) only if a long fragment was taken from *s* and then SLRs were sequenced from the fragment with barcode *b*. Typically, for each correct barcode *b* we can find multiple reads that align in different positions across *s*. Contrary, for incorrect barcodes we expect zero or only a few reads that are aligned locally only in some repetitive regions close to each other. In order to distinguish correct and incorrect barcodes, we use the number of reads with barcode *b* aligned to the segment, and the distance between the leftmost and rightmost reads aligned to the segment. In our experiments, if a barcode has at least three read pairs aligned to segment *s* and the distance between the leftmost and rightmost reads is greater than 5*kbp*, we include it in B(s), otherwise, it is discarded.

**Figure 2.**
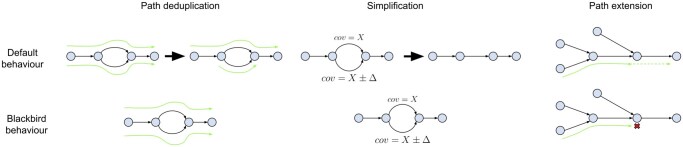
SPAdes adaptation to human segmented data. Left: SPAdes’ path deduplication procedure is relaxed, so if a pair of paths have small differences, both paths are kept for SV calling. Middle: SPAdes simplification procedures are relaxed to keep heterozygous SVs. Right: SPAdes PathExtend procedure is changed to account for sudden coverage drops, which can form tips in the assembly graph and lead to erroneous deletion calls.

We gather all reads with barcodes in B(s) aligned to segment *s* by scanning the position-sorted input BAM file and storing them as paired reads whenever possible or single reads. Then we add reads from M(B(s)). Next, the gathered reads are assembled using our modified version of SPAdes assembler (Prjibelski *et al.* 2020). We adapted SPAdes to a very specific data type we want to assemble (see Fig. 2).

### 2.3 Adapting SPAdes for human SV detection

SPAdes was originally designed for bacterial isolate assembly, which differs significantly from human genome assembly. Key differences include:

- **Ploidy.** The human genome is diploid, leading to assembly graph structures such as bulges that represent heterozygous SNVs. Unlike bacterial assemblies, where low-frequency variations are often removed as contamination or sequencing errors, human assemblies benefit from preserving these variants to correctly phase the genome.
- Structural Complexity. For SV detection, maintaining alternative paths in the assembly graph is crucial, whereas bacterial assemblies prioritize a single consensus sequence.

To adapt SPAdes for human SV calling, we introduced three key modifications:

- Relaxed Path Deduplication. In bacterial assemblies, highly similar paths (e.g., those threading different sides of a bulge) are deduplicated to reduce redundancy. For SV detection, we disable this deduplication, ensuring that structural variations are retained for analysis.
- Modified Graph Simplification. SPAdes aggressively removes tips and bulges in bacterial assemblies to minimize duplication. We relaxed these procedures to retain diploid variation, essential for identifying heterozygous SVs.
- Refined Path Extension Rules. Assembly-based SV detection methods can generate false deletions in regions with low sequencing coverage. To mitigate this, we introduced a topology-based refinement that prevents contig extension in cases where a coverage drop might lead to an erroneous deletion call.

These changes improve SPAdes’ handling of human genome data, preserving essential SV-related structures in the assembly (Fig. 2).

In (Rahman and Medvedev 2022), the authors present examples of misassemblies in assembly graphs that appear error-free. These misassembles are often attributed to imperfect genome coverage resulting from sequencing biases. Even a local drop in coverage can lead to a misassembly event, particularly in repetitive regions. Unfortunately, SLR data frequently exhibit sequencing biases that create coverage gaps across the genome (see Supplementary Fig. S2).

Mapping-based methods can typically distinguish coverage drops from actual deletions, as there are no split-read or discordant paired-end read signatures associated with the coverage drop. However, for assembly-based methods like SVABa or Blackbird, coverage drops in repetitive regions may result in false-positive deletion calls.

To mitigate this issue, we implemented a new topology rule for contig extension (Fig. 2). In scenarios with perfect coverage of the target genome, it is safe to extend a path using an edge without alternatives. However, coverage drops can create long tips, which we do not expect to encounter in human sequencing data. We modified this rule to check for the presence of long tips in the vicinity and refrain from extending the contig in such cases.

In hybrid mode, long-reads are also used during the local assembly step. We add those long-reads that are aligned to the segment to the assembly. Path extension procedure for hybrid assembly was not altered, but we added long-read sequences to the graph construction procedure. Another improvement was to use long-reads to close the gaps not only between tips but also between arbitrary parts of the graph. In this gap-closing step, we limit the number of long-reads to 100 reads per segment to reduce the computational cost (see Supplementary Fig. S3).

### 2.4 Identification of structural variants

Assembled contigs are aligned to the reference genome using minimap2. Insertions and deletions are called using the CIGAR (Concise Idiosyncratic Gapped Alignment Report) string, which encodes the alignment operations between the contig and the reference genome. Due to the diploid nature of the genome, some events might be called multiple times. We implemented a deduplication procedure that removes events that have the same breakpoint location and length as some other event. Resulting insertions and deletions are sorted and stored in a variant call format (VCF) file. Resulting insertions and deletions are sorted and stored in a VCF file, a standard tab-delimited format for representing genomic variants, including their positions, reference and alternative alleles, and additional annotations.

### 2.5 Software availability

Blackbird is freely available at https://github.com/1dayac/Blackbird.

## 3 Results

Benchmarking SV detection tools is quite challenging due to the large number of available tools and the difficulty of selecting a subset of relevant ones. For comparison, we picked Manta (Chen *et al.* 2016) since according to (Kosugi *et al.* 2019), a recent comprehensive evaluation of SV detection tools, this is the best short-read method overall on the real data. As Blackbird is an assembly-based method, with a focus on smaller and medium-sized events, we also selected SvABA (Wala *et al.* 2018) as a representative comparison. LEVIATHAN (Morisse *et al.* 2021) is an SLR SV detection tool that uses barcode information, while LongRanger is an SLR aligner that calls deletions during the read alignment step. LongRanger is not able to call insertions, and it was only assessed for deletions in our evaluations.

To the best of our knowledge, there are no existing SV callers that are able to work in this hybrid setting. We decided to compare Blackbird in hybrid mode against the state-of-the-art long-read SV callers—Sniffles2 and PBSV.

We compared Blackbird, long-read, and short-read tools performance on the simulated dataset, and the HG002 dataset that has a well-established benchmark callset of insertions and deletions (Zook et al. 2020).

We tested Blackbird in SLR mode against Manta, SvABA, and LEVIATHAN, and in hybrid mode against Sniffles2 and PBSV. Specifications of tested tools can be found in “Supplementary text A: Tool specifications”. For each tool, we compare calls with insertion/deletion sizes between 50 and 10 000 bp. For SV matching and comparison, we used Truvari (English *et al.* 2022), which allows us to compare a callset obtained by one of the tools to the reference callset. First, we divide target and reference VCF files into separate deletions and insertions/duplication VCF files. These files are compared against each other using Truvari. We exclude events shorter than 50 bp because all assembly-based methods have low precision for short events since genome assembly is prone to mismatches and small insertions and deletions. Precision and recall are the metrics we use to evaluate methods. The exact command we used to run Truvari can be found in “Supplementary text B: Running Truvari.”

### 3.1 The simulated dataset

#### 3.1.1 Hybrid mode

**Figure 3.**
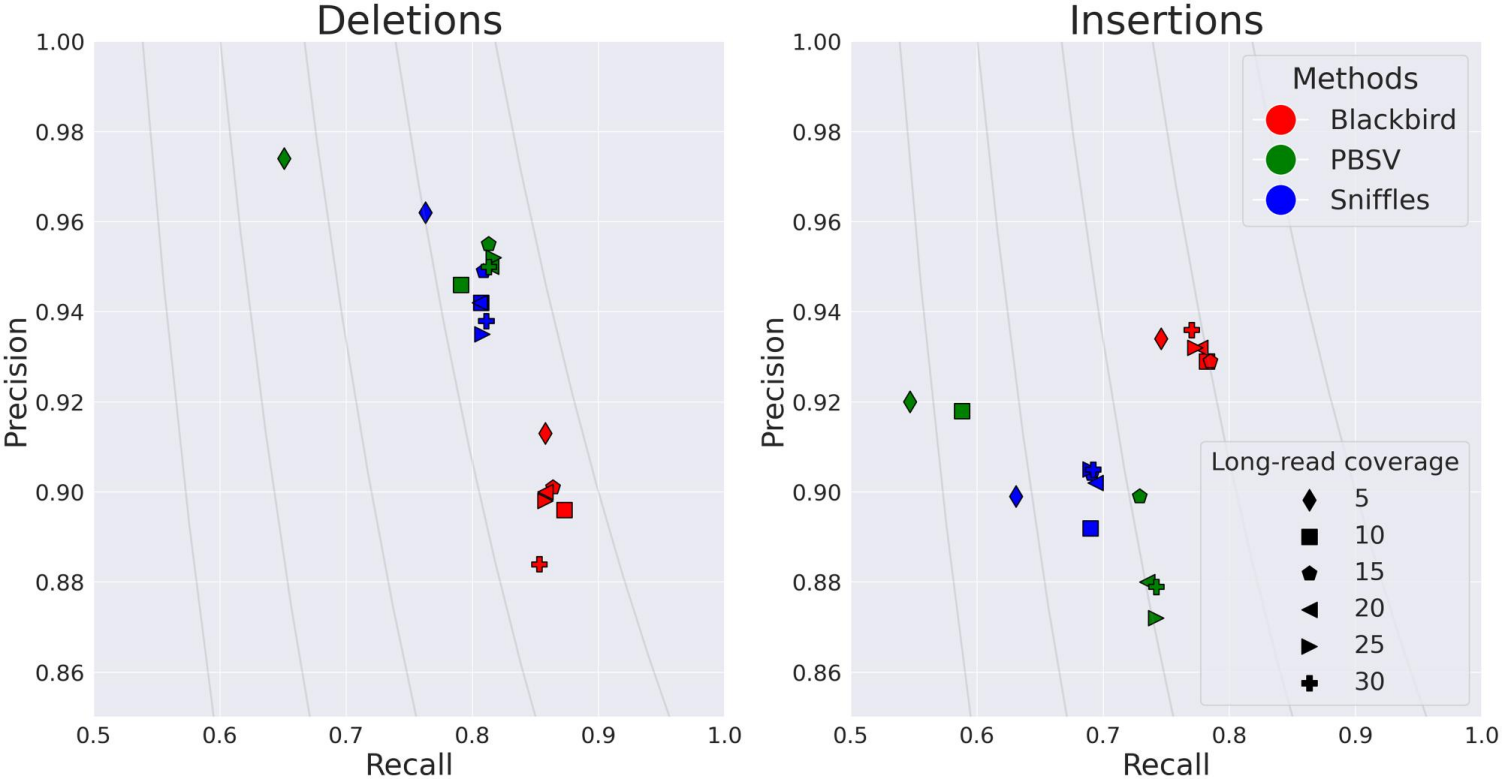
Results for the hybrid simulated dataset. Blackbird is able to achieve the highest precision and recall for insertions, and better recall for deletions. While Blackbird has lower precision for deletions than competitors, it is able to achieve better F1-scores. Curves are F1 curves.

In order to evaluate the performance of Blackbird within a fully controlled environment, we first created an SLR simulated dataset. We added insertions and deletions from (Huddleston and Eichler 2016) to chromosome 1 of the GRCh38 reference genome. Such an approach allows us to test performance on various insertion and deletion repeat families, such as L1, *Alu*, and STR. We inserted 1437 events with a total length of 799 kbp. Additional information about these events can be found in “Supplementary text C: Simulated SV events.” We then simulated a 37× SLR dataset using LRSIM (Luo *et al.* 2017) with default read simulation parameters. We aligned reads to chromosome 1 of the GRCh38 genome using LongRanger. Additionally, we simulated PacBio Hi-Fi long reads with 9000–12 000 read lengths and 0.01–0.001 error rate per read, at coverage levels of 5, 10, 15, 20, 25, and 30, using bbmap’s randomreads module (Bushnell 2014). We tested Blackbird in hybrid mode against state-of-the-art long-read SV callers PBSV and Sniffles2. We compare the results of the methods above with the list of ground-truth SVs. We count a predicted SV as correct if there exists a corresponding SV in the ground truth set of the same type and their position on the reference is not further than 500 bp away from each other. Blackbird was able to achieve 85–87% recall for deletions, 75%–77% recall for insertions, and precision in the 90%–93% range for all datasets (Fig. 3). Using these results, we can argue, that SV callers have saturation property, such that at some point additional long-read coverage adds only incremental or no improvement to the results. Blackbird’s results in hybrid mode are superior than the results in SLR mode even with 5× long-read coverage, but results do not improve with increased long-read coverage. Sniffles2 performance saturates at 10× long-read coverage and PBSV does not saturate at lower than 10× rate but saturates at 15× rate. It can be seen that Blackbird in hybrid mode with 5× long-read coverage is superior to PBSV and Sniffles2 with 5× coverage, and comparable (slightly better for insertions and slightly worse for deletions) with Sniffles2, PBSV with 15× coverage.

These findings lead to the conclusion that Blackbird can achieve the same SV calling quality with lower long-read coverage. Thus, Blackbird can be a cost-effective alternative or can be used when high coverage with long reads is not readily available.

#### 3.1.2 SLR mode

**Figure 4.**
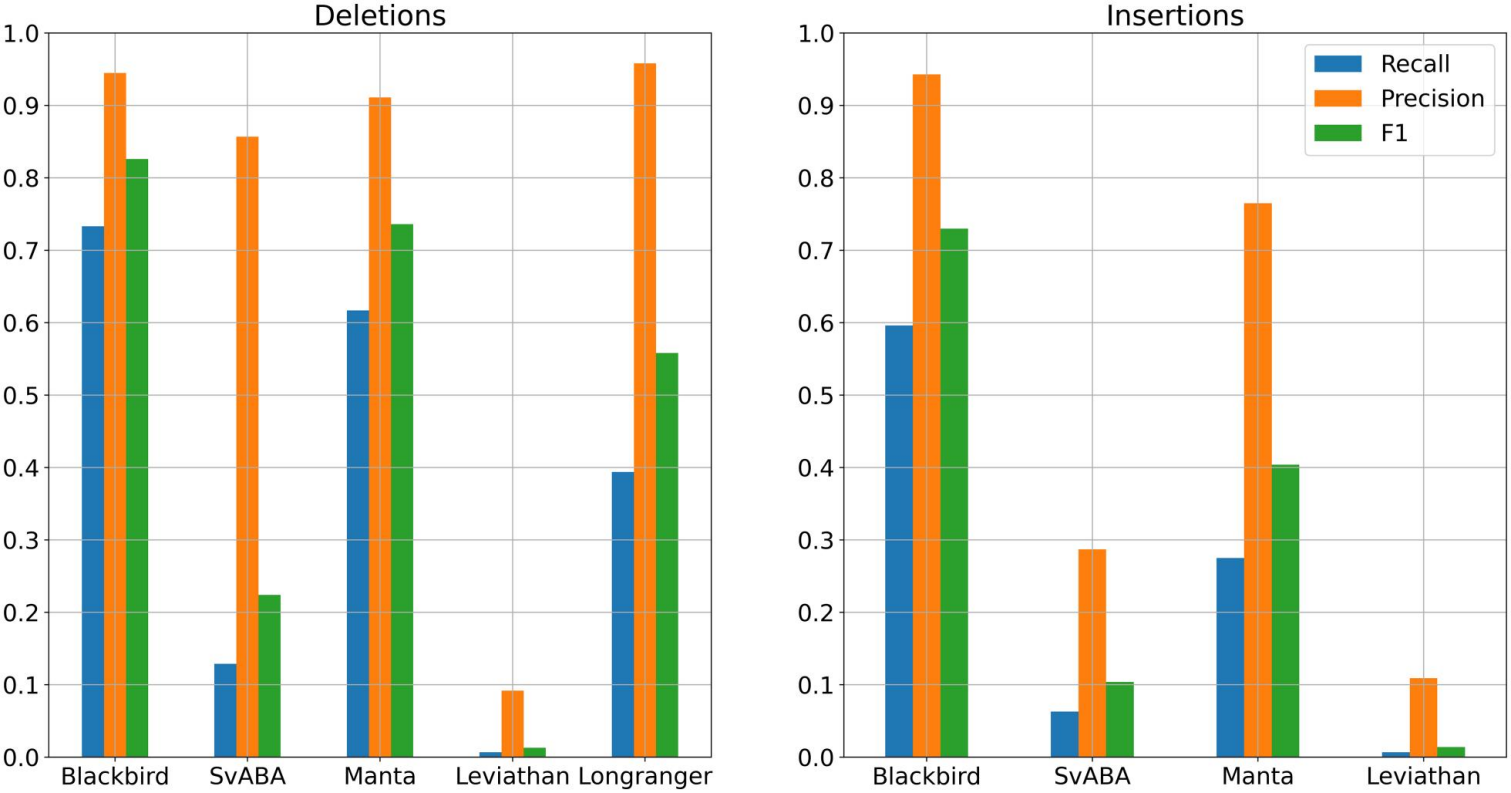
Results for the SLR simulated dataset. Blackbird is able to achieve the highest recall and F1-score both for insertions and deletions. These results show that Blackbird is able to call insertions with much higher recall that other short-read SV calling tools.

We evaluated the performance of Blackbird, LongRanger, Manta, SvABA, and LEVIATHAN on the same simulated SLR-only dataset. We show the precision and recall of each method in Fig. 4.

On the simulated dataset, Blackbird achieved the best over all result with the highest sensitivity and F1 score for both insertions (58% and 0.71, respectively) and deletions (72% and 0.81, respectively). Additionally, Blackbird achieved the highest precision for insertions (94%) and managed to obtain precision for deletions at 94% level, slightly behind LongRanger (95%). Therefore, Blackbird achieves the best average results on the simulated dataset. Interestingly, the performance of all short-read/SLR tools is limited by a majority (54%) of STR SVs in the simulated data. Blackbird is also able to call the most of *Alu* repeats and half of L1 repeats (see Table S1).

### 3.2 The HG002 dataset

**Figure 5.**
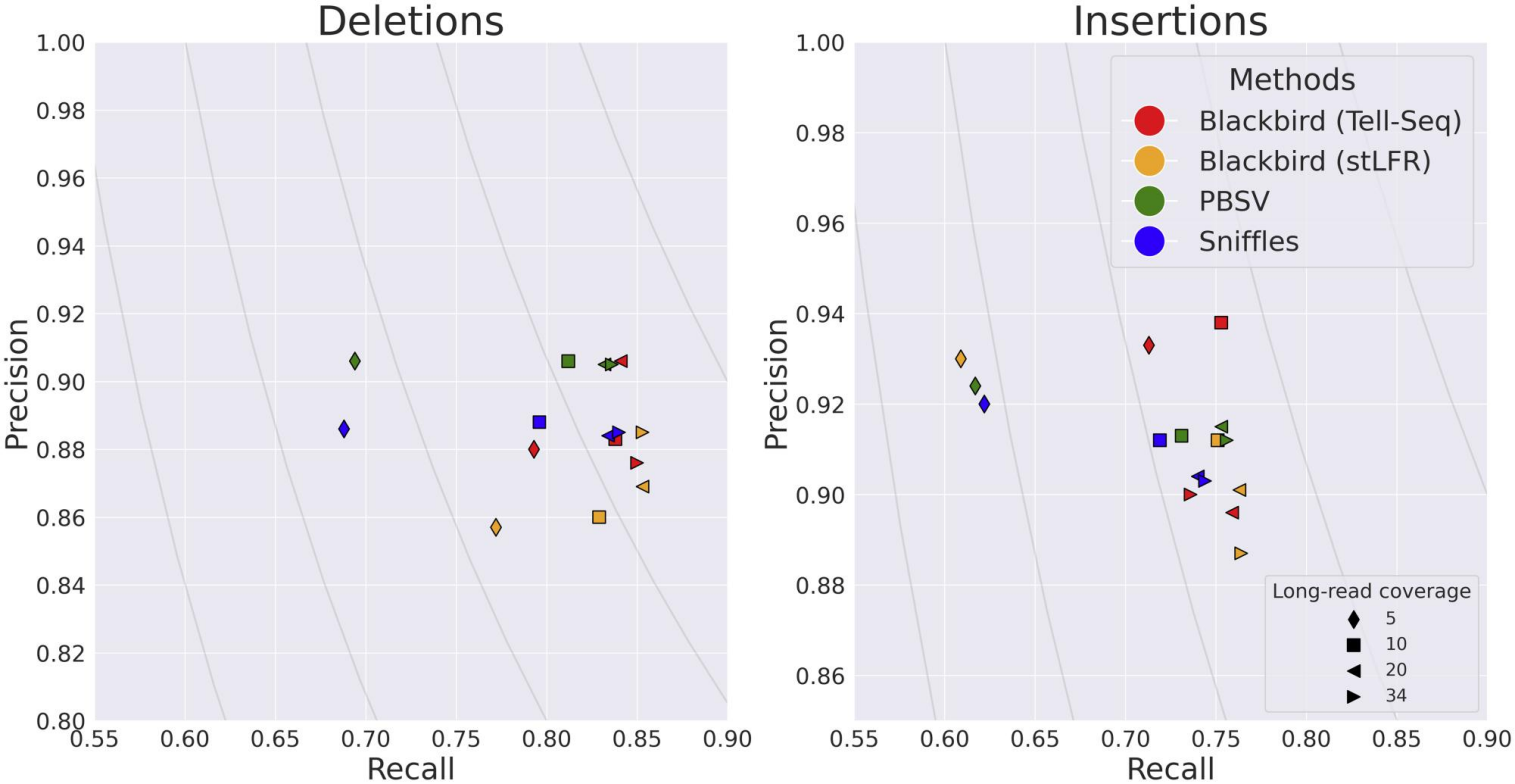
Results for the HG002 hybrid dataset. Left: Deletions. Blackbird results saturate with lower long-read coverage. E.g. Blackbird on TELL-Seq data and stLFR data with 5× long-read coverage appears close to the runs with the higher coverage on the plot. PBSV and Sniffles2 have a 12–15% drop in recall when long-read coverage is equal to 5×. Right: Insertions. Blackbird on TELL-Seq data performs similarly to the deletions run. However, Blackbird on stLFR data with 5× long-read coverage performs similarly to Sniffles2 and PBSV. Also, Blackbird is able to achieve the highest recall for insertions and deletions when the coverage is high. Curves are F1 curves.

**Figure 6.**
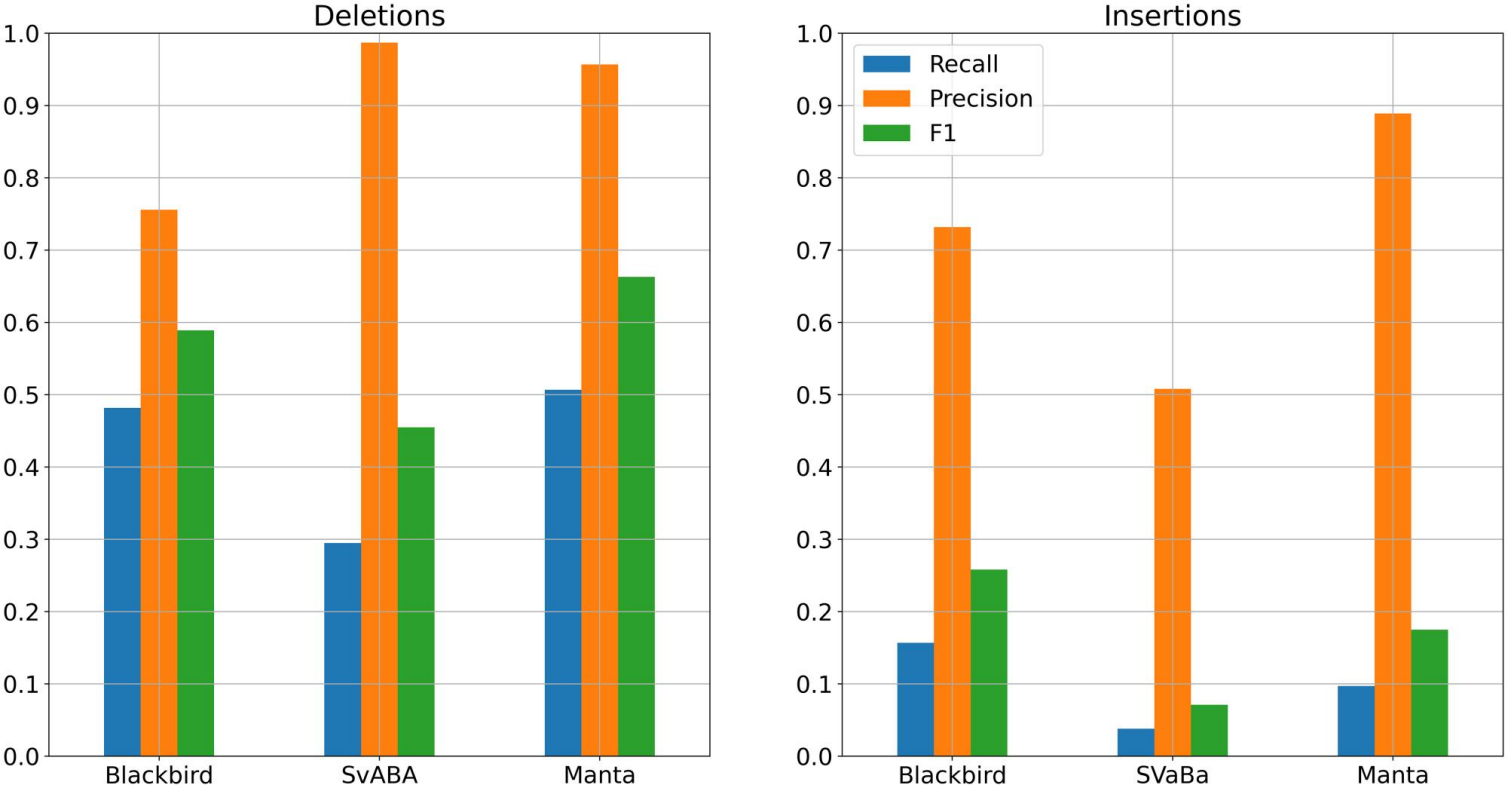
Results for the SLR HG002 dataset. Left: Deletions. Manta achieved better recall and precision for deletions, though Blackbird recall is only 2% lower. Right: Insertions. Blackbird is able to double the recall for the insertions on the HG002 dataset compared to the Manta. Some of the insertions found by Blackbird were longer than 1 kbp.

The HG002 genome has been extensively characterized by the Genome in a Bottle (GIAB) consortium with a curated insertion and deletion benchmark callset (Zook *et al.* 2020) for the GRCh37 reference genome (HG002_SVs_Tier1_v0 0.6). We used UST TELL-Seq data with 42× coverage and 169 bp read length with a 20% addition of shorter reads and stLFR data with 35× coverage and 100 bp read length as benchmark SLR datasets. These two technologies are among the most popular SLR technologies publicly available. We aligned SLR reads to the reference genome using LongRanger that was modified to accept TELL-Seq and stLFR barcodes. For PacBio Hi-Fi data, we used a dataset from the PacBio website with an average read length of around 16 500 bp for 10× and 5× coverage, and T2T PacBio Hi-Fi data with an average read length of around 12 500 bp for 34× and 20× coverage. In order to get 5× and 20× datasets, we downsampled higher-covered PacBio datasets. For SLR mode comparison we used the stLFR dataset mentioned above. The results are presented in Figs. 5 and 6. In the SLR mode comparison, Blackbird demonstrates recall comparable to Manta for deletions while achieving the highest recall for insertions, all while maintaining reasonable precision. This highlights the advantage of SLR technologies over standard short-read sequencing in detecting long insertions. Specifically, while short-read sequencing struggles to call insertions exceeding twice the fragment length due to the lack of contiguous sequence representation, SLR technologies provide long-range connectivity, enabling more accurate detection of insertions over 1000 bp. In hybrid mode comparison, PBSV and Sniffles2 have a stable precision rate, and recall increases with increased coverage. However, it is clear that on the HG002 dataset results saturate only with 10–20× long-read coverage. When long-read coverage drops to 5× rate, precision drops by 10%–15% for both insertions and deletions compared to 10× coverage. On the other hand, when TELL-Seq data is used, Blackbird’s recall drops only by 4% for deletions and 2% for insertions. At 10× coverage, Blackbird also performs better in terms of recall compared to Sniffles2 and PBSV. Blackbird with stLFR data performs worse than Blackbird with TELL-Seq data when the long-read coverage is within 5–10× range but achieves the best recall compared to all other methods on higher long-read coverage. These results show that Blackbird, especially with TELL-Seq data, is able to achieve saturation earlier than Sniffles2 and PBSV. They also suggest that for the SV detection problem, there is no need to go deeper than 20×, as the improvements are only incremental at higher coverage.

We explored what kind of sequences are called with Blackbird in hybrid mode with TELL-Seq and long reads at 5× coverage but are not called by Sniffles2 and PBSV at 5× long-read coverage (see Supplementary Figure S4). In order to do that, we extracted SV sequences from the GIAB callset, ran them through RepeatMasker, and counted the amount of called sequences for each reported repeat type. Results show that there is no designated repeat type that benefited exclusively. Additional Blackbird calls are scattered across different repeat types and non-repetitive sequences uniformly, excluding simple repeats, where the number of calls is almost similar. Information about how zygosity and long-read coverage impact recall can be found in “Supplementary text D: Zygosity and Long-Read Coverage in SV Calling.”

#### 3.2.1 Dataset of challenging medically relevant genes

Another source of curated SV calls is the dataset of challenging medically relevant autosomal genes (Wagner *et al.* 2022) for the HG002 genome. This dataset contains around 200 SVs, which are reported to be more challenging on average than the variants from the GIAB dataset. We performed additional benchmarking using this dataset. The results are presented in “Supplementary Text E: Dataset of Medically Relevant Genes.” While Blackbird shows lower performance compared to Sniffles2 and PBSV for this subset of relatively complex events, it still achieves good precision and recall (86% and 71% for 5× long-read coverage).

### 3.3 Running time analysis

We conducted experiments to assess the running time and memory usage of Blackbird on Intel Xeon Gold 6230 2.1–3.9 GHz processors with 32 cores, using the HG002 dataset. For our SLR dataset, we used the 42× stLFR dataset mentioned earlier, and for our long-read dataset, we used the 34× PacBio Hi-Fi dataset.

Blackbird, when operating in SLR mode, was expectedly slower than the standard short-read methods SvABA or Manta. Specifically, on the HG002 dataset, Blackbird required 6 hours and 12 Gb of memory, while SvABA finished in 65 min and 11.5 Gb and Manta was able to complete the task in just 15 min and 6.5 Gb.

When running in hybrid mode, Blackbird took 11 h to finish and utilized 117 Gb of memory. In comparison, Sniffles2 required only 3 min and 0.6 Gb, while PBSV needed 1 h 19 min and 27G. Despite these performance differences, we believe that Blackbird’s increased sensitivity with low-coverage long reads makes it a viable alternative for SV calling.

## 4 Conclusion

We introduced Blackbird, a tool designed to address the need for accurate SV detection when only moderate amounts of long-read sequencing data are available. High-coverage long-read sequencing remains prohibitively expensive for large-scale studies, and although short-read sequencing is cost-effective, it struggles to resolve complex SVs, particularly insertions. Blackbird fills an important niche by enabling sensitive SV discovery at reduced coverage, leveraging SLR and low-coverage long-read data. Sequencing cost is an important factor in selecting an appropriate SV detection approach. Short-read sequencing (e.g. Illumina) remains the most cost-effective option, but its limitations in detecting certain types of structural variants, particularly long insertions, significantly reduce its utility. Long-read sequencing technologies (e.g. PacBio Hi-Fi and Oxford Nanopore) provide superior resolution but are considerably more expensive per gigabase, making them less accessible for large-scale studies. Also, obtaining high-coverage long-read sequencing data can be difficult or impractical in clinical settings where sample material is limited (e.g. small tumor biopsies) or in retrospective analyses of archived samples with degraded or exhausted DNA material. SLR technologies, such as 10× Genomics or BGI stLFR, offer a balance between cost and long-range information. Currently, SLR sequencing is marginally more expensive than short-read sequencing, whereas long-read sequencing remains several times more costly than short-read approaches. Blackbird effectively integrates long-read and SLR sequencing data, reducing the need for additional long-read sequencing and lowering overall costs.

While the current implementation of Blackbird is limited to insertions and deletions, we plan to implement modules for inversions in the future. While Blackbird is not capable of working with larger events (e.g. translocations), there are available tools that can call that events effectively. We also plan to improve the performance of our algorithm on diploid data, such as distinguishing between homozygous and heterozygous variants and adding variant phasing. At the moment Blackbird operates on a contig scale, which makes haplotyping and homozygous/heterozygous classification challenging. However, our integration with SPAdes genome assembler makes it possible to use an assembly graph structure to better classify heterozygous variants.

Blackbird assembles each genomic segment, even regions where read-pair or split-read signal cannot be found. On the other hand, potentially, Blackbird can be transformed into a reference-assisted SLR assembly tool. There are no existing algorithms that solve this problem, and Blackbird’s performance is already better than existing *de novo* assembly tools such as Supernova. Future work can also include running time optimization. A major bottleneck is caused by the need to constantly search for read mates in a large indexed BAM file. BAM index provides semi-random access by position; its running time is highly dependent on the local coverage around the mate’s position. For some segments, the naive solution, which requires a separate query for each mate can be too slow.

## Supplementary Material

vbaf151_Supplementary_Data

## Data Availability

**Tell-Seq Data** is available under SRX7264481 accession in SRA. **stLFR Data:** Link to stLFR Data **PacBio Hi-Fi Data:** Link to PacBio Hi-Fi Data **ONT Data** is available under PRJNA200694 BioProject
